# Multicenter Study of Secukinumab Survival and Safety in Spondyloarthritis and Psoriatic Arthritis: SEcukinumab in Cantabria and ASTURias Study

**DOI:** 10.3389/fmed.2021.679009

**Published:** 2021-05-26

**Authors:** Sara Alonso, Ignacio Villa, Sabela Fernández, José L. Martín, Lilyan Charca, Marina Pino, Leyre Riancho, Isla Morante, Monserrat Santos, Anahy Brandy, Elena Aurrecoechea, Loreto Carmona, Rubén Queiro

**Affiliations:** ^1^Rheumatology Division, Hospital Universitario Central de Asturias, Oviedo, Spain; ^2^Rheumatology Division, Hospital Sierrallana, Torrelavega, Spain; ^3^Rheumatology Division, Hospital Universitario de Cabueñes, Gijón, Spain; ^4^Instituto de Salud Musculoesquelética, Madrid, Spain; ^5^Instituto de Investigación Sanitaria del Principado de Asturias (ISPA) Translational Immunology Division, Hospital Universitario Central de Asturias (HUCA), Oviedo, Spain

**Keywords:** secukinumab, safety, survival, spondyloarthritis, psoriatic arthritis, comorbidities

## Abstract

**Objectives:** We aimed to evaluate the drug retention rate and safety of secukinumab (SEC) in patients with axial spondyloarthritis (AxSpA) and psoriatic arthritis (PsA) in a real clinical setting.

**Methods:** This multicenter retrospective observational study included all AxSpA and PsA patients who received at least one dose of SEC. Adverse events (AE) and the drug retention rate were the main study outcomes. Drug survival was analyzed by Kaplan-Meier curves while predictive factors of discontinuation were evaluated using a Cox regression analysis. The weight of these associations was estimated by hazard ratio (HR) values.

**Results:** We included 154 patients (59 PsA and 95 AxSpA). Mean disease duration was 6.5 years (IQR 2-8). Sixty-one percent of patients were treated with two or more biologics prior to SEC. The 1 and 2-year retention rates for SEC were 66 and 43%, respectively. The main causes of discontinuation were inefficacy (59%) and AE (36%). The factors associated with lower risk of discontinuation were male gender (HR 0.54, 95% CI 0.38-0.78 *p* = 0.001), obesity (HR 0.53, 95% CI 0.30-0.93 *p* = 0.027), hypertension (HR 0.55, 95% CI 0.30-0.93 *p* = 0.008), and diabetes (HR 0.42 95% CI 0.18-0.99 *p* = 0.047) while number of previous biologics and depression were predictors of discontinuation (HR 1.18, 95% CI 1.04-1.34 *p* = 0.011 and HR 2.53, 95% CI 1.61-3.96 *p* < 0.001).

**Conclusions:** SEC showed a good retention rate in a population previously exposed to several biological therapies. As a novelty, cardiometabolic comorbidities were associated with better drug survival.

## Introduction

Secukinumab (SEC) is a human monoclonal antibody (IgG1) directed against IL-17A, approved for the treatment of plaque psoriasis (1), psoriatic arthritis (PsA) and axial spondyloarthritis (AxSpA) (2, 3). The safety profile observed for SEC during clinical development is generally not different from other biological therapies including among others, infections, neutropenia, and hypersensitivity reactions. Data from randomized controlled clinical trials (RCTs) and post-marketing surveillance have shown that SEC has a favorable safety profile over long-term treatment (4), even with fewer adverse events and low frequency of treatment discontinuation (2, 4–6). Compared to placebo, SEC has been described to increase the incidence of upper respiratory tract infections and an increased incidence of mucocutaneous Candida infections, middle ear infections, and herpes simplex infections has also been observed compared to placebo (7). Some severe cases and exacerbations of Crohn's disease have also been described (8), so caution is recommended with its use. In terms of routine clinical practice, studies show a safety profile similar to that previously reported in RCTs and their long-term extension studies (9–12), but information from real-world evidence studies is still scarce. Data on survival of biological therapy in PsA from the DANBIO registry showed a median survival of the first TNF inhibitor (TNFi) being 2.2 years and the second and third TNFi being 1.3 and 1.1 years, respectively. Switchers were more frequently women, had a shorter disease duration, a higher median Health Assessment Questionnaire (HAQ) score, DAS28 and fatigue and pain scores (on a VAS), and had more swollen and tender joints compared to non-switchers when they started the first TNFi (13). Likewise, the NOR-DMAR registry performed an analysis in patients who switched from one TNFi to another, finding that survival of the second TNFi was only 3 years in 36% (14). However, we currently have no consistent data on long-term survival of SEC in patients with AxSpA and PsA in routine clinical practice. Efficacy, the number of previous treatments, specific comorbidity and perhaps obesity and smoking are factors that may determine SEC survival.

Therefore, we aimed to evaluate the safety of SEC in actual clinical practice, as well as to study drug retention and causes for discontinuation, and to evaluate factors associated with SEC suspension in patients diagnosed with PsA or AxSpA who receive or have received such therapy.

## Methods

We designed a multicenter retrospective longitudinal observational study. The project adhered to the postulates of the Declaration of Helsinki and its extensions as well as to the rules of good clinical practice and General Data Protection Regulation. The ethics review board of Sierrallana Hospital approved the study and exempted the participants from informed consent due to the retrospective nature of the study (EPA-OD, code: HUC- SEC-2019-01).

### Study Population

Eligible subjects were all adult patients with a diagnosis of AxSpA (age range: 27-77 years) by the Assessment of Spondyloarthritis International Society (ASAS) classification criteria (15) or PsA (age range: 24-81 years) by the Classification Criteria for Psoriatic Arthritis (CASPAR) (16) who received at least one dose of SEC in three hospitals from northern Spain.

The two primary outcomes were safety and drug survival. Safety was analyzed by reviewing the clinical charts from the date of initiation of SEC, as well as the hospital admission records; the following were specifically checked: (1) Infections (type, microorganism, location and whether it was accompanied by bacteremia); (2) neoplasms (type, location, and stage); (3) Events located or affecting any other organs or systems. Drug survival (in months) was defined as the time from the start of SEC to the last dose administered if discontinued or to the last dose administered if lost to follow-up. The reason for suspension was also collected.

The following secondary variables were collected: descriptive and explanatory variables related to treatment, disease, and comorbidities, indication (AxSpA, PsA), dose of SEC administered, corticosteroids, tobacco (active, ex-smoker, never), age, years evolution of the illness, sex, enthesitis, dactylitis, diabetes mellitus, hypertension, obesity, dyslipemia, depression, chronic obstructive pulmonary disease (COPD), major adverse cardiovascular events (MACE), ischemic cardiopathy, renal and hepatic insufficiency, biological treatment line (1st line, 2nd, 3rd, 4th...), primary or secondary failure, previous serious AEs, previous disease-modifying antirheumatic drugs (DMARDs) (yes/no and concomitant), BASDAI, BASFI, ASDAS, MDA, BSA, ESR, CRP, hemoglobin (baseline and at 6 and 12 months).

### Statistical Analysis

The sample was described in terms of the distribution of the descriptive variables by summary statistics. The rate of AEs was estimated in total, by severity and by type of event. The denominator used was the total number of patients^*^years of follow-up. Survival was analyzed using Kaplan-Meier curves and the hazard ratio was used as a measure of the association. Multivariate Cox regression was used to analyze the effect of the explanatory variables on survival. Potential confounding variables were previous and concomitant treatments, and comorbidities.

A random sample of 68 individuals was deemed sufficient to estimate, with 95% confidence and a precision of ±5% units, a population rate of AE expected to be around 15%, and a percentage of replacements of 5%.

## Results

154 patients were included, 59 with PsA (38%) and 95 with AxSpA (62%), with a mean age of disease onset of 49 years (SD ± 11), being 55% men. The median disease duration was 6.5 years (IQR 2-8). Table 1 shows a description of the study population, by diagnosis and total.

**Table 1:** Disease characteristics of the study population.

**Characteristic**	**Psoriatic arthritis *n* = 59**	**Axial spondyloarthritis *n* = 95**	**Total *n* = 154**
Age, mean (SD)	51 (12)	47 (10)	49 (11)
Male	27 (46)	58 (61)	85 (55)
Disease duration, m (SD)	7 (8)	6 (5)	6 (7)
Number of previous biologics, median (IQR)	3 (2)	3 (2)	3 (2)
csDMARD prior to SEC	39 (66)	15 (16)	54 (36)
Type of csDMARD			
Methotrexate	34 (58)	10 (11)	44 (29)
Leflunomide	9 (15)	3 (3)	12 (8)
Sulfasalazine	1 (2)	5 (5)	6 (4)
Other	3 (5)	1 (1)	4 (3)
Glucocorticoids	18 (31)	10 (11)	28 (19)
Secukinumab dose 300 mg	34 (57)	11 (12)	45 (29)
Obesity (BMI > 30)	16 (27)	10 (11)	26 (17)
Smoker	15 (26)	29 (31)	44 (29)
Hypertension	17 (29)	18 (19)	35 (23)
Dyslipidemia	22 (37)	19 (20)	41 (27)
Diabetes	9 (15)	3 (3)	12 (8)
COPD	1	-	1
Cardiovascular disease^*^	1	2	3 (2)
Ischemic heart disease	4 (7)	3 (3)	7 (5)
Depression	10 (17)	17 (18)	27 (18)
Chronic Kidney Disease	2	-	2
Hepatic failure	1	2	3 (2)

*Cells include n (%) unless otherwise indicated*.

* *Myocardial infarction or cerebrovascular event*.

*SD, standard deviation; IQR, interquartile range; csDMARD, conventional synthetic disease modifying anti-rheumatic drug; BMI, body mass index; COPD, Chronic obstructive pulmonary disease*.

The population was largely refractory to biological therapy: SEC was the first line of treatment in 13 patients (8%), the second line in 46 (30%), the third line in 54 (35%) and subsequent lines in 41 (27%).

**Figure 1:**
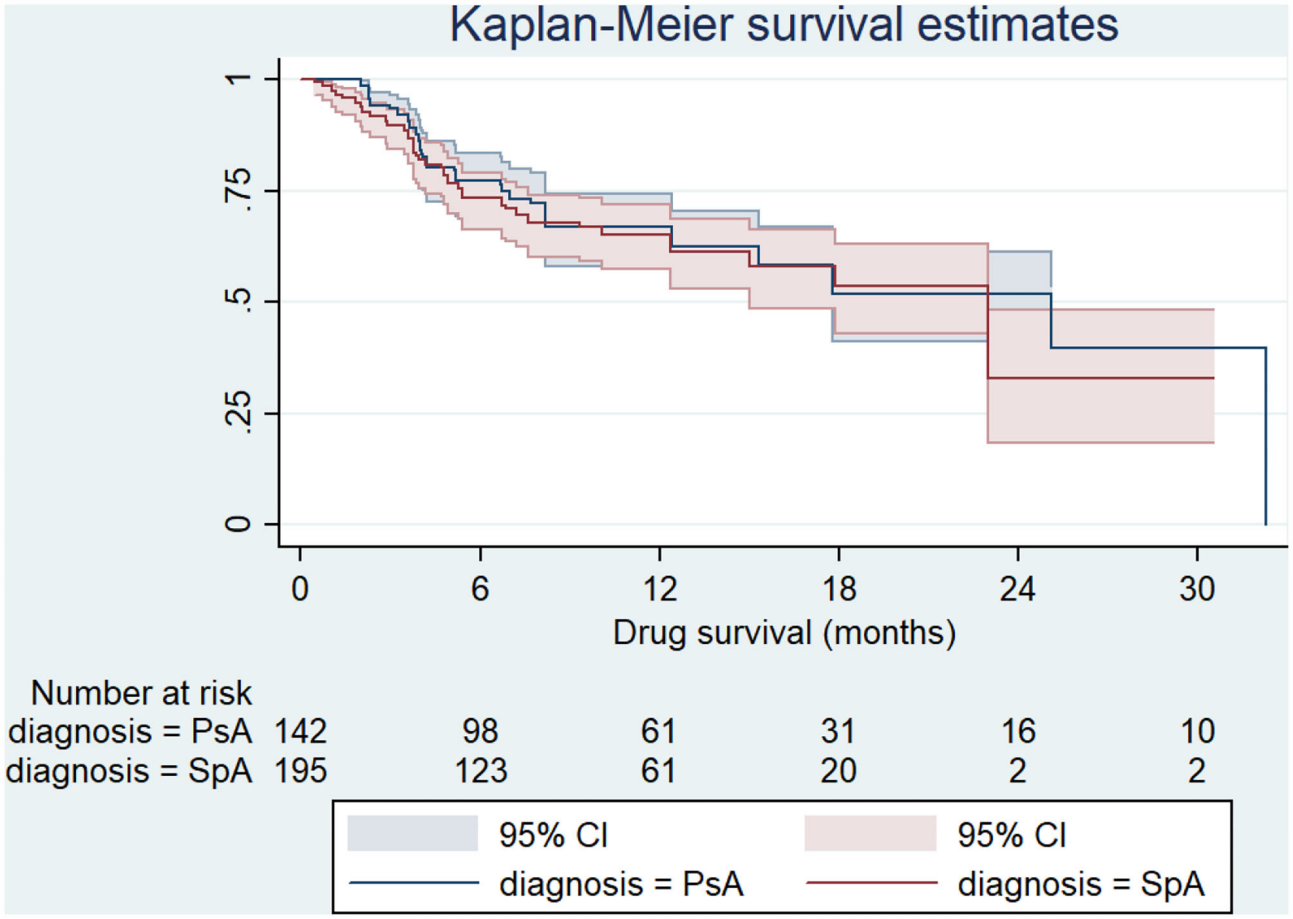
Survival curve of secukinumab by disease types. PsA, Psoriatic arthritis; SpA, Spondyloarthritis.

The median survival of SEC was 23 months (IQR 5-32), with a 1-year retention rate of 66% and a 2-year retention rate of 43%. No differences were found between AxSpA and PsA (log-rank *p* 0.526) (Figure 1).

The main cause of SEC discontinuation was inefficacy (59%) followed by AEs (23 cases, 36%). Most patients who discontinued due to AEs (71%) did so during the first 6 months of treatment. The rate of discontinuation due to AE was 6.4 per 1,000 persons-years (95% CI: 4.1-9.7). The most frequent AE were gastrointestinal (nausea, vomiting, and abdominal pain, including two cases of Crohn's disease), cutaneous (mainly generalized rash, pruritus, and papulo-nodular lesions), and infections (mostly upper respiratory tract). One major cardiovascular event was collected, and a neoplasm was diagnosed in two patients during treatment. Crohn's disease was diagnosed in two patients during the exposure. Table 2 shows a description of the AEs identified.

**Table 2:** Description of adverse events collected.

**Adverse event**	***n* (%)**	**Withdrawal**
Disorders of the blood and lymphatic system	0 (0)	-
Heart disorders	1 (0.6)	1/1
Congenital, familial and genetic disorders	0 (0)	-
Disorders of the ear and vestibular maze	1 (0.6)	1/1
Endocrine disorders	0 (0)	-
Eye disorders	0 (0)	-
Gastrointestinal disorders	6 (3.8)	6/6
General symptoms and local injection site reactions	0 (0)	-
Hepatobiliary disorders	0 (0)	-
Immune system disorders	0 (0)	-
Traumatic injuries, intoxications and complications of therapeutic procedures	0 (0)	-
Disorders of metabolism and nutrition	0 (0)	-
Musculoskeletal and connective tissue disorders	0 (0)	-
Disorders of the nervous system	0 (0)	-
Pregnancy, puerperium and perinatal diseases	0 (0)	-
Psychiatric disorders	1 (0.6)	1/1
Kidney and urinary disorders	0 (0)	-
Reproductive and breast disorders	0 (0)	-
Respiratory, thoracic and mediastinal disorders	1 (0.6)	1/1
Disorders of the skin and subcutaneous tissue	5 (3)	5/5
Social circumstances	0 (0)	-
Vascular disorders	0 (0)	-
Infections	5 (3)	4/5
Neoplasms	2 (1)	2/2

*Cells include n (%)*.

*Withdrawal: No. patients in whom the adverse event led to discontinuation of the drug*.

**Figure 2:**
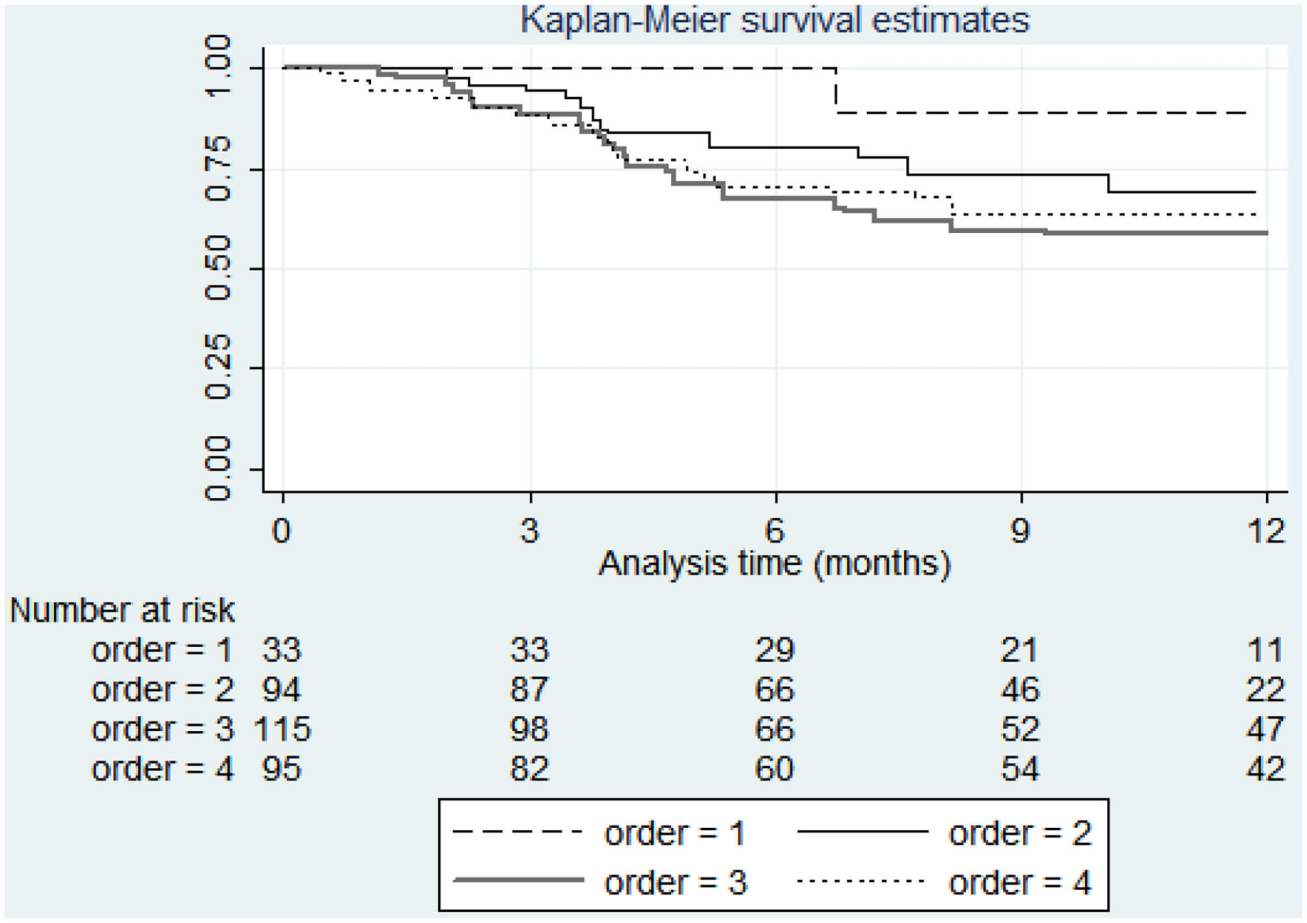
Survival curve of secukinumab by biologic order.

**Figure 3:**
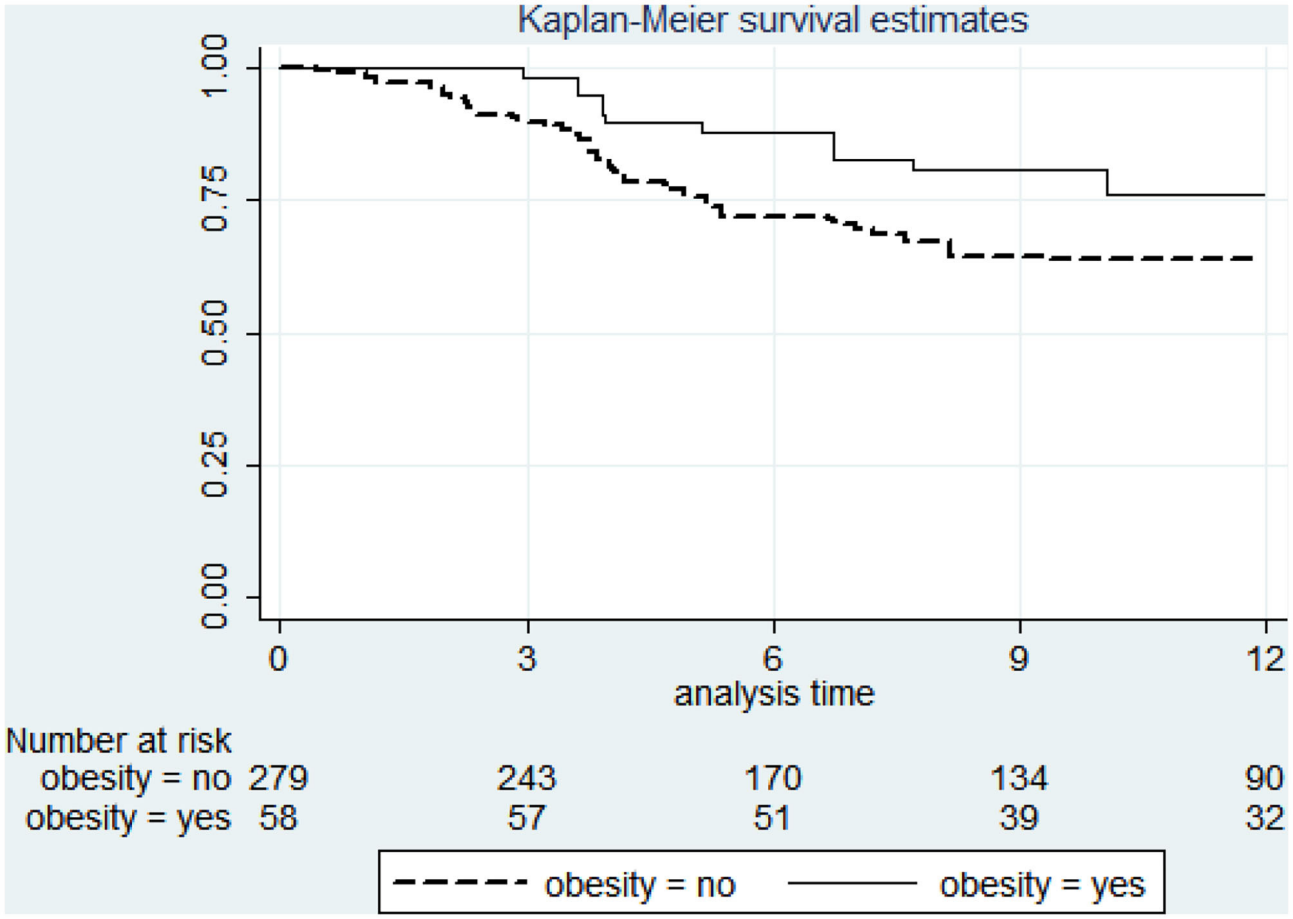
Survival curve of secukinumab by obesity.

The factors associated with lower risk of discontinuation were male gender (HR 0.54, 95% CI 0.38-0.78 *p* = 0.001), obesity (HR 0.53, 95% CI 0.30-0.93 *p* = 0.027), hypertension (HR 0.55, 95% CI 0.30-0.93 *p* = 0.008), and diabetes (HR 0.42 95% CI 0.18-0.99 *p* = 0.047) while number of previous biologics and depression were predictors of discontinuation (HR 1.18, 95% CI 1.04-1.34 *p* = 0.011 and HR 2.53, 95% CI 1.61-3.96 *p* < 0.001). The survival by treatment line (biologic order) and by obesity are shown in Figures 2 and 3. Table 3 shows bivariable and multivariable survival analysis.

**Table 3:** Bivariable and multivariable survival analysis.

	**HR (95% CI)**	
**Characteristic**	**Bivariable**	**Multivariable**
Age, per year	1.00 (0.99 - 1.02)	
Male sex	0.77 (0.54 - 1.09)	0.54 (0.38 - 0.78)
Disease duration, per year	0.97 (0.95 - 1.00)	0.97 (0.94 - 1.00)
**Biologic order**		
First biologic	Ref.	
Second	2.49 (0.97-6.38)	3.62 (1.39-9.44)
Third	2.77 (1.10-6.98)	4.25 (1.66-10.93)
Fourth or more	2.53 (0.99-6.44)	5.09 (1.93-13.44)
csDMARD prior to Sec	0.75 (0.52-1.09)	
Glucocorticoids	2.00 (0.80-1.81)	
Obesity (BMI>30)	0.49 (0.30-0.82)	0.53 (0.30-0.93)
Smoker	1.09 (0.87-1.35)	
Hypertension	0.51 (0.34-0.77)	0.55 (0.35-0.85)
Dyslipidemia	0.87 (0.60-1.27)	
Diabetes	0.29 (0.13-0.63)	0.42 (0.18-0.99)
Cardiovascular disease^*^	4.63 (0.64-33.39)	
Ischemic heart disease	0.64 (0.26-1.58)	
Depression	2.10 (1.38-3.21)	2.53 (1.61-3.96)
Kidney failure	1.40 (0.56-3.49)	
Hepatic failure	1.09 (0.44-2.68)	

* *Myocardial infarction or cerebrovascular event*.

## Discussion

In this clinical practice study conducted in 154 patients with AxSpA and PsA, treatment with SEC showed a 66% 1-year retention rate in a population largely refractory to biological therapy irrespective of the disease and the number of biologics previously received. The main cause of discontinuation in our study was lack of efficacy while AEs leading to discontinuation of the drug occurred mainly in the first 6 months of treatment. Factors associated with longer SEC survival were male gender, obesity, hypertension and diabetes. Number of previous biologics and depression were identified as negative predictors for drug survival.

Discontinuation or switching of biological agents in inflammatory arthritis is quite common. Switchers receiving their second TNFi agent usually have considerably poorer responses compared with non-switchers (17), so switching to other biological disease-modifying antirheumatic drugs (DMARDs) with different mechanisms of action may be a better therapeutic strategy alternative (18). Among the main factors related to reduced TNFi survival different studies have found female sex, shorter disease duration, number or previous biologics, older age, current smoking, and comorbidities (13, 19–21). Other studies showed a better overall drug survival in patients with higher C-reactive protein (CRP) levels and patients replacing the first TNFi as a result of adverse events (21–23). The effect of concomitant methotrexate (MTX) on TNFi survival varies among registry studies, with similar TNFi persistence on combination therapy and monotherapy in the CORRONA registry (24), a trend toward longer drug survival in the SSATG study (22) and the DANBIO study (25) and significantly longer drug survival in the NOR-DMARD study (20). It is unclear at this time whether combination therapy with conventional DMARDs could influence SEC survival, and our data do not allow us to make any claims about this either, so further studies are needed.

Patients who start SEC retain the drug in a large percentage for at least 12 months according to clinical trials (5), but long term data regarding SEC survival in daily clinical practice are limited. Also, patients in clinical trials may be subject to selection bias as they are recruited on the basis of different clinical or disease characteristics, comorbidities and/or concomitant drugs. Factors that may affect SEC survival have not yet been established with certainty. In another Spanish observational study conducted in real clinical practice, Pinto et al. analyzed the efficacy and safety of SEC in 76 patients with peripheral PsA who started treatment in a 1-year period. 71% of patients had received at least one biological treatment before SEC. In line with our study, the retention rate of SEC at 12 months was lower in the group previously treated with biologics (81.5 vs. 90.9%) (26). Other real-life studies both in patients with PsA and AxSpA, had shown similar results with an ever decreasing SEC survival rate in patients who had previously received biological therapy (11, 26–28). We found only one study in real-life in which the survival rate of SEC was not related to the number of previous biologics. In the study by Gentileschi et al. evaluating the long-term efficacy of SEC in only 39 patients with radiographic and non-radiographic AxSpA, it was found an overall 2-year retention rate of 78.2% with no significant differences between biologic-naïve and anti-TNF-failure patients (29). Regarding other possible factors associated with SEC survival, Chimenti et al. published a real-life, prospective observational study on 169 consecutive patients (39 with ankylosing spondylitis and 130 with PsA) treated with SEC over a 1-year period. Most patients had received at least one biological drug (79%), and in line with our data they found higher persistence rate in male patients than female (30).

Obesity has been related to a higher risk of immune-mediated inflammatory diseases with an obesity range prevalence of 10–50% among these patients (31). High body weight has been associated with accelerated clearance resulting in lower trough concentrations of TNF blockers (32). Furthermore, visceral fat has been shown to independently contribute to an increased systemic inflammatory load (33). Obesity is associated to a poor prognosis in patients with rheumatic diseases, especially psoriasis and PsA (34). Obese patients with PsA are less likely to achieve minimal disease activity (MDA), more likely to discontinue treatment and also show lower skin clearance rate. This effect appears to be proportional to body mass index (BMI) and, in fact, weight reduction improves response to treatment (4, 35). In a recent meta-analysis, obesity was associated with 60% higher odds of failure to an index TNFi therapy in patients with rheumatoid arthritis, AxSpA, and psoriatic disease (36).

On the other hand, the therapeutic response to SEC tends to be poor in obese psoriasis patients (37). However, we found that obesity, and other components of metabolic syndrome, were predictors of longer survival for SEC therapy. Therefore, SEC could be a good therapeutic choice in obese patients with AxSpA and PsA as opposed to TNFi agents. In line with our data, Pantano et al. analyzed 100 PsA patients treated with EC. Patients were divided into two groups based on their BMI. After 6 months of SEC, changes of the Disease Activity Index for Psoriatic Arthritis (DAPSA) were inversely related to BMI values (38). Analysis of IL-17 serum levels showed significantly higher serum levels of this cytokine among obese patients. Also, Tiberio et al. described two cases of obese patients with psoriasis and PsA effectively treated with SEC (39). Therefore, SEC seems to be an efficacious drug irrespective of body weight. PASI 75, PASI 90, and PASI 100 response rates were high across weight quartiles and were maintained through week 52 in the pooled analysis of phase III trials with SEC. Moreover, SEC 300 mg dose demonstrated consistently greater benefit than the 150 mg dose across weight quartiles (40). Data from the CLEAR study showed that SEC 300 mg had a significantly higher efficacy (PASI 90 at week 16) than Ustekinumab 90 mg in patients with a body weight over 100 kg (41). All these data indicate that obesity may be one of the most relevant clinical factors driving the choice of SEC over other drugs in patients with AxSpA and PsA. IL-17 has been associated with insulin resistance and obesity in patients with psoriatic disease (42). Moreover, IL-17-deficient mice are characterized by enhanced insulin sensitivity and increased glucose uptake. In human co-culture experiments, macrophage-derived IL-1β was shown to enhance IL-17 production. Since macrophages do express IL-17 receptors, there might exist a positive and paracrine feedback loop that enhances local visceral adipose tissue inflammation (42). In line with these findings, our study shows that patients with a cardiometabolic inflammatory profile (obesity, diabetes, hypertension, arthritis) show better SEC survival, thus giving IL17 a central role in the pathogenesis of this inflammatory phenotype.

In our study we identified depression as a predictor of poor survival of SEC. According to the EULAR recommendations for the management of PsA and AxSpA, comorbidities such as depression should be considered (43, 44). Depression affects nearly 20% of patients with PsA (45) and SpAs and is associated with increased disease activity (higher BASDAI, ASDAS, DAPSA, and CDAI), worse functional impairment (higher BASFI, BASMI, and PGA), poor prognosis and greater non-adherence (45). Different studies have shown that patients with major depression have overexpressed levels of pro-inflammatory cytokines, acute phase reactants, and chemokines. Thus, IL-6, IL-17, and TNF levels are higher in patients with depression compared with healthy controls. These alterations may partly explain the correlation between higher inflammation, depressive symptoms and pain, the depression-pain syndrome, and the worse impact of the disease reported by patients on PROs (46, 47). Regarding fibromyalgia, although some of our patients met this profile, most of them fell into the category of patients with depression, so this aspect was not assessed independently.

Treatment with biological therapy, mitigating pain and reducing inflammation, may suggest a beneficial effect on the control of depressive symptoms and therefore a better response and increased survival of these therapies in patients with PsA or AxSpA. However, the present study identified depression as a risk factor for reduced survival among patients with PsA or AxSpA treated with SEC (HR 2.52, 95% CI 1.61-3.95 *p* = 0.000). Our results are in line with Danish (48) and British cohort (19) studies which included 1,750 and 566 PsA patients treated with TNFi therapy and with a Canadian cohort of 825 patients with ankylosing spondylitis and PsA (49). In all these cohorts, baseline depression negatively affected the response to TNFi therapy and was correlated with higher baseline disease activity and shorter TNFi persistence. Our study showed similar results of drug retention with an anti-IL17A therapy.

Our study has some limitations, which deserve to be discussed. First, we acknowledge that the sample size was relatively small and that the study was performed within an ethnically homogeneous population being cared for in various centers in north Spain, and therefore, these results may not be generalizable. Second, the collection of data in a retrospective manner may carry a certain risk of bias due to the lack of standardization in data collection. Unfortunately, we did not make a distinction between radiographic and non-radiographic AxSpA. This distinction is relevant because as Lopalco et al. demonstrated, the effectiveness of TNFi seems to be lower in non-radiographic AxSpA patients than in those with radiographic disease (50). The strength of our study is the interest of real clinical practice studies to complement the results of clinical trials, providing valuable data regarding the overall safety, efficacy and survival of a drug in heterogeneous patient populations usually with co-morbidities not registered in RCTs. In addition, data of SEC survival on Spanish population are still scarce.

In conclusion, in this study of real clinical practice, SEC showed a 66% retention rate at 1 year in a population mostly refractory to biological therapy. Treatment persistence has been optimal even in third line treatment, independent of the underlying disease, and obesity does not seem a marker of poor treatment response. The implications of our findings should be replicated in larger cohorts.

## Data Availability Statement

The raw data supporting the conclusions of this article will be made available by the authors, without undue reservation.

## Ethics Statement

Ethical approval for this study was obtained from the Sierrallana Hospital of Torrelavega, Spain (HUC- SEC-2019-01). Written informed consent for participation was not required for this study in accordance with the national legislation and the institutional requirements.

## Author Contributions

IV, SA, SF, EA, and RQ: study design, data management, analysis, verification, interpretation, and writing. LoC: study design, analysis, interpretation, and writing. JM, LiC, MP, LR, IM, MS, and AB: data management, analysis, and interpretation. All authors contributed to the article and approved the submitted version.

## Conflict of Interest

IV consultant of: UCB, Speakers bureau: Novartis, MSD, Lilly, JM Grant/research support from: AbbVie, Pfizer, Janssen, and Celgene, Speakers bureau: Pfizer and Lilly, LR Grant/research support from: Yes, Speakers bureau: Yes, LoC Grant/research support from: Novartis Farmaceutica, SA, Pfizer, S.L.U., Merck Sharp & Dohme España, S.A., Roche Farma, S.A, Sanofi Aventis, AbbVie Spain, S.L.U., and Laboratorios Gebro Pharma, SA (All trhough institution). The remaining authors declare that the research was conducted in the absence of any commercial or financial relationships that could be construed as a potential conflict of interest.
